# Improvement of Fatty Acid Profile and Studio of Rheological and Technological Characteristics in Breads Supplemented with Flaxseed, Soybean, and Wheat Bran Flours

**DOI:** 10.1155/2014/401981

**Published:** 2014-11-12

**Authors:** Mariana B. Osuna, María A. Judis, Ana M. Romero, Carmen M. Avallone, Nora C. Bertola

**Affiliations:** ^1^Universidad Nacional del Chaco Austral, Departamento de Ciencias Básicas y Aplicadas, Laboratorio de Industrias Alimentarias I, Cte. Fernández N°755, Chaco, 3700 Presidencia Roque Sáenz Peña, Argentina; ^2^Centro de Investigación y Desarrollo en Criotecnología de Alimentos (CIDCA), CONICET, Facultad de Ciencias Exactas, Buenos Aires, 1900 La Plata, Argentina

## Abstract

Functional breads constitute an interesting alternative as vehicle of new essential fatty acids sources. The aim of this study was to improve the fatty acids (FA) profile of bakery products, producing breads with low saturated fatty acid (SFA) content and with high polyunsaturated fatty acid (PUFA) content, through partial substitution of wheat flour by other ingredients (soy flour, flax flour, and wheat bran) and to analyze the effect of this change on the technological, rheological, and sensorial characteristics of breads. Flaxseed flour (FF), soybeans flour (SF), or wheat bran (WB) was used to replace 50, 100, and 150 g kg^−1^ of wheat flour (WF) in breads. FF or SF produced a decrease in monounsaturated and SFA and an increase of PUFA in these breads. Furthermore, breads replaced with FF presented considerable increase in the content of n3 FA, while, SF or WB contributed to rise of linoleic and oleic FA, respectively. The substitution percentage increase of FF, SF, or WB to formulation produced changes in the colour, rheological, textural, and technological characteristics of breads. This replacement resulted in improved lipid profile, being breads with 50 g kg^−1^ SF, the better acceptance, baking features, and enhanced fatty acid profile.

## 1. Introduction

The Argentine Food Code (CAA) [1] establishes that the bread with fat is the product prepared in the same way of the French bread with addition of no less than 4% edible fats, being the beef fat the most used. This bread is the most consumed in the northeast region of Argentina and is produced in traditional bakeries. The mean daily bread consumption is 190 g per capita [2]. Therefore, from the point of view of cardiovascular diseases, the consumption of bakery products is not recommended [3]. These products are associated with the presence of saturated fatty acids (SFA) and* trans*-fatty acids (TFA), and it is well known that these fatty acids cause an increase in plasma cholesterol, mainly LDL cholesterol, and in the total HDL cholesterol ratio, with a subsequent increase in cardiovascular risk [4]. The total lipid intake and the consumption ratio of polyunsaturated fatty acids (PUFA) long chain n6/n3 has increased significantly in the Western diet in recent decades [5]. In human diet, the most characteristic SFA is palmitic acid, followed by stearic and myristic acids. Unlike PUFA, palmitic and myristic acids are also considered responsible for an increase in total and LDL cholesterol levels in serum, hence increasing coronary risk, and oleic fatty acid is responsible for raising HDL cholesterol levels in a manner comparable with unsaturated fats [6]. n3 fatty acids have important roles in the modulation and prevention of human diseases, particularly coronary heart disease [7]. The advances in different scientific areas, the increasing chronic disease rates, and the fact that consumers recognize the relationship between diet and health led to the development of functional foods by food industry [8].

According to American Dietetic Association [9] functional foods have a potentially beneficial effect on health. In order to reduce the saturated fat content of processed foods, the food industry in developed countries has gradually replaced animal fat by vegetable fat, which provides oils with a high content of unsaturated fats [10]. Therefore, the bakery industry has two major challenges: first, to make low-fat products having organoleptic characteristics similar to the original products and, second, to adapt their formulations and production processes to using fats with better nutritional characteristics.

Fortunately, bakery products are ideal for providing a variety of healthy ingredients [11]. Soybeans are unique among the legumes because they are a concentrated source of isoflavones. Isoflavones have weak estrogenic properties and the isoflavone genistein influences signal transduction. Soy foods and isoflavones have received considerable attention for their potential role in preventing and treating cancer and osteoporosis [12]. Flaxseed (*Linum usitatissimum*, Linn.; Linaceae) is an interesting raw material for food applications within the emerging concept of functional foods [13]. Flaxseed contains good amount of *α*-linolenic acid (LNA), omega-3 fatty acid, protein, dietary fiber, lignan, and specifically secoisolariciresinol diglucoside (SDG). Several studies reveal that these components work well for nutritional benefit in human being. LNA is beneficial for infant brain development, reducing blood lipids and cardiovascular diseases. Flaxseed dietary fiber exhibits positive effect to reduce constipation, to keep better bowel movement, and as hypocholesterolemic agent. SDG have antioxidant activity and free oxygen radical scavenging activity. Consequently, it may have anticancer property [14]. Whole-grain-based diets have been suggested to reduce the incidence of cardiovascular disease and colon cancer. Phenolic compounds, most of which are present in the wheat bran, may be one of the factors contributing to whole-grain health benefits [15]. Wheat bran is an excellent source of dietary natural antioxidants and phenolic acids and may contribute to total dietary carotenoids and tocopherols [16]. In recent years, considerable interest has been generated in the development and consumption of foods enriched with various healthy ingredients.

There is enough literature available on nutritional characterization of flaxseed, soybeans, chia, wheat bran, and others grains [10–15] and their utilization in processed foods [16–19]. Nevertheless, knowledge and comparison about the effect of supplementation of wheat flour with flaxseed flour, soy and wheat bran on the rheological properties, and the baking characteristics of bread are scant.

Hence present work focuses on use of brown flaxseed flour, soybeans flour, and wheat bran as a functional ingredient in bread. The aim of this study was to improve the fatty acids profile of bakery products, producing breads with low SFA content and with high PUFA content, through partial substitution (50, 100, and 150 g kg^−1^) of wheat flour by others ingredients (soy flour, flax flour, and wheat bran) and to analyze the effect of this change on the technological, rheological, and sensorial characteristics of breads.

## 2. Materials and Methods

### 2.1. Raw Materials

Conditioned wheat flour (WF) (commercial mixture of 000 and 0000, Florencia, Argentina), whole soybean flour (SF), whole flaxseed flour (FF), wheat bran (WB), and bovine fat (BF) (Friar, Argentina) were used for the preparation of the breads. Compressed yeast (*Saccharomyces cerevisiae*, Calsa, Argentina) was used as leavening agent. The moisture content of yeast was 715 g kg^−1^.

### 2.2. Baking Test

A straight dough method was used for preparation of bread [20]. The basic formula for the control bread was as follows: WF (1 kg), compressed yeast (40 g), bovine fat (40 g), sodium chloride (20 g), and tap water up to optimum absorption (55 g kg^−1^). Flaxseed flour (FF) or soybeans flour (SF) or wheat bran (WB) was used to replace 50, 100, and 150 g kg^−1^ of wheat flour (WF). The different steps were performed at laboratory scale. The ingredients were mixed and kneaded to an optimum consistency in a rapid mixer (Zonda, Buenos Aires, Argentina) for 7 min, and then they were put to rest in bulk for 15 min. After this period, the dough was laminated into the sheeter (RD, Buenos Aires, Argentina) and allowed to stand for 15 min again. Then, the dough was divided into portions of 400 g, was rounded manually, and was allowed to stand for 15 min. They were cut into portions of 200 g and the loaves were armed and were placed into aluminum pans (24.5 × 6.5 cm). The pans were placed in a proving cabinet at 35°C and 85% RH for 90 min. The fermented dough was baked in an electric oven (ZONDA, Buenos Aires, Argentina) for 15 min at 180 ± 5°C. After baking, the bread samples were removed from the pans and cooled to room temperature (23 ± 1°C). Then, the bread was packed in polyethylene bags for their subsequent analysis. The experiments were done in triplicate.

### 2.3. Fatty Acids Composition

Total lipids were extracted using the Bligh and Dyer method [21]. Fatty acid composition was determined after methylation using an Agilent (model 6850A HP, Agilent Technologies Inc., CA, USA) gas chromatography with a Supelco 2340 capillary column of 60 m and 0.25 mm internal diameter according to AOAC N° 969.33 (AOAC, 1990). The temperature of the injector and detector was kept at 250°C. The injected volume was 1.0 *μ*L. The carrier gas was helium at 0.6 *µ*L min^−1^. The split ratio used was 1 : 100. The temperature of the column was kept at 140°C for 5 minutes, raised to 240°C at 4°C/min, and maintained at 240°C for 10 minutes. Fatty acids were identified by comparison of their retention times with those of authentic standards (Supelco 37 Components FAME Mixture, Bellefonte, PA) and reported as g kg^−1^ of total fatty acids determined. Results were expressed as relative quantities of saturated (SFA), monounsaturated (MUFA), and polyunsaturated fatty acids (PUFA), PUFA/SFA ratio, and n6/n3 ratio.

### 2.4. Technological Parameters

Technological parameters were carried out according to American Association of Cereal Chemists (AACC) [22]. Moisture contents of bread were determined by measuring their weight loss upon drying in an oven at 130°C still reaching constant weight. Samples of bread were analyzed and results were expressed in grams of water per 100 g of wet solid (AACC [22] 44-19). The loaf specific volume (SV) was determinate in all samples of the loaves. The loaf volume of the bread was determined by flaxseed displacement (AACC [22] 10-05) and was calculated by dividing the loaf volume by the weight (cm^3^ g^−1^). All determinations of the technological characteristics of bread were performed in triplicate.

### 2.5. Breadcrumb Texture Profile Analysis

Texture measurements were performed on cylinder (20 × 20 mm thick), cut out from the central part of the three replicated loaves for each formulation, 15 h after baking. On average, six measurements per slice were made. The bread cylinders were compressed using a textural analyzer (CT3, Brookfield, EEUU) equipped with TA25/1000 cylinder probe (50.8 mm diameter, Acrylic, Rad 0.35–0.43 mm, Brookfield, EEUU). For texture profile analysis (TPA), samples were compressed to 50% of their original height. The uniaxial compression test was performed in two successive cycles, using 0.5 mm s^−1^ as the speed test [23]. The typical texture profile analysis parameters were determined from the Force-Distances curves and calculated by the software: hardness (N), cohesiveness (adimensional), gumminess (N), springiness (mm), and chewiness (mJ) [24].

### 2.6. Color Analysis

The color of crumb and crust of breads was determined with a Thermo Fisher scientific spectrophotometer with an integrating sphere (Hunter Associates Laboratory, Inc., Reston, VA) and the results were expressed in accordance with the CIELab system with reference to illuminant D65 and a visual angle of 10°. The parameters determined were *L* ^*^ (lightness), *a* ^*^ (Redness), and *b* ^*^ (yellowness). Three replicates were made.

### 2.7. Stress Relaxation of Crumb Bread

Stress relaxation of breads was measured according to the method proposed by Sozer et al. [25] with some modifications. Stress relaxation measurements were performed on cylinder (20 × 20 mm thick), cut out from the central part of the three replicated loaves for each formulation, 15 h after baking. The stress relaxation test was executed by using a TA25/1000 cylinder probe (50.8 mm diameter, Acrylic, Rad 0.35–0.43 mm, Brookfield, EEUU). The sample was deformed in the compression to a constant strain of 30% with test speed of 0.5 mm/s. The residual force was continuously recorded as a function of time for 300 s. The experimental data were modeled by using nonlinear regression in Sigma-Plot 8.0 software (Systat Software Inc., San Jose, CA, USA) based on a least square algorithm. The stress relaxation data were analyzed by using a Maxwell model.

### 2.8. Sensorial Analysis

The formulations with control similar technological and rheological characteristics were selected to evaluate the general acceptance. A preference test was carried out with one hundred and five untrained panelists. The first three slices of the ends of the bread were not used, and the remaining slices (20 g) were cut into six parts and offered to the judges. A five-point hedonic scale: (5) like very much, (4) like moderately, (3) neither like nor dislike, (2) dislike moderately, and (1) dislike very much, was used.

### 2.9. Statistics

Results were expressed as the mean values of at least 3 replications. The correlations between relaxation and texture parameters were evaluated by the method of Pearson and values of correlation coefficient (*r*) were reported. Multiple sample comparison of the means and Tukey least significant differences were applied to establish statistical significant differences between treatments. All statistical analyses were carried out with the Infostat software (National University Cordoba, Cordoba, Argentina) and differences were considered significant at *P* < 0.05.

## 3. Results and Discussion

### 3.1. Fatty Acid Composition

The fatty acids composition of the raw ingredients (WF, FF, SF, WB, and BF) is given in Table 1. These analyses showed clearly that beef tallow fat presented larger amount of SFA, being almost 60% of its composition. The SFA were stearic, palmitic, and, to a lesser extent, miristic and heptadecanoic. Also, BF presented MU (oleic) and PUFA (linoleic and linolenic). The vaccenic fatty acid was detected in BF. These results were in agreement with da Cunha et al. [26], who found a similar composition of fatty acids in BF.

WF and WB had higher content SFA. Therefore, palmitic fatty acid was larger in WF and WB than SF and FF, while the stearic fatty acid content was lower. Menteş et al. [27] also found that the content of palmitic fatty acid was around 18% in WF, 7% in FF, being these results somewhat higher than those our study results. Also, that stearic acid was less in WF compared with FF. Research has shown that SFA have adverse effect on plasma lipids and their consumption is associated with a high cardiovascular risk, so the recommendation is to reduce consumption of these fatty acids. However, not all SFA behave the same way. The stearic acid (18 : 0) is exception. Stearic acid has low gastrointestinal absorption and does not adversely modify the plasma lipids, which is considered “neutral” for cardiovascular health [28]. Furthermore, the amount of monounsaturated was higher in SF. MU are represented by oleic acid (C18: 1 n9), which is present predominantly in olive oil, and in other oils such as canola (rapeseed with low erucic fatty acid), high oleic sunflower oil; nuts and animal fat (especially in pigs fat fed with acorns) [29]. The oleic fatty acid in the WF was lower than in the other flours, being higher in SF and WB. The fatty acids of the n6 family were predominant in PUFA content of WF, WB, and SF. Also, FF had the highest content of PUFA. Linoleic acid content of the WF and WB presented higher quantities than SF and FF. These results were similar to Menteş et al. [27], Simbalista et al. [30], and Santos Calderelli et al. [31], to the content of linoleic of FF and WF. The level of LNA of WB and WF was less than the values found in FF and SF, the flax being more than 12 times higher than those of wheat. Our results were according to Morrison [32], who found the similar composition of fatty acids of WB. Menteş et al. [27] found that the proportion of LNA in the distribution of flaxseed fatty acids was higher (58.14%). The high content of PUFA of the FF limits its use in food due to oxidation causing rancid flavors on the same. Linolenic acid contains a total of three double bonds, which make the fatty acid twenty-five times more susceptible to rancidity, whereas linoleic acid has a total of two double bonds and has approximately half the reactive rate [33]. WB had a fatty acid composition rich in linoleic acid and oleic fatty acid. It also had saturated fatty acids, palmitic and to a lesser extent, stearic. None of the analyzed flour presented detectable amounts of elaidic acid.

According to nutritionists, a well-balanced diet should maintain the proportion of fatty acids n6/n3 in a 5 to 10 range or a lower relation [34]. The ratio n6/n3 of different flours was in accord with the WHO recommendations [34], except for FF. Due to high content of n-3 fatty acids, FF presented a very small n6/n3 ratio. In addition, the PUFA/SFA ratio of all analyzed flours was greater than 0.45, as recommended by WHO [34].

Tables 2 and 3 show the relative quantities of the identified fatty acids, as well as total contents of SFA, MU, PUFA, n3, n6, and n9 (g kg^−1^ of total fatty acids) and relationship of PUFA /SFA and n6/n3 of loaves replaced with SF, FF, and WB (50, 100, and 150 g kg^−1^) and control. All analyzed parameters presented significant differences with the control (*P* < 0.05).* trans*-fatty acids were detected in all formulations, though the relative percentages of these fatty acids decreased when the level substitution with SF, FF, and WB was increased. Loaves with FF presented the lowest amount of* trans*-fatty acids. Therefore, the content of myristic, palmitic, stearic, and oleic fatty acids also decreased with increasing the percentage of substitution in breads with FF and SF as compared with the control. Consequently, the SFA and MU content decreased in these breads. The loaves with 150 g kg^−1^ FF presented the largest decrease of SFA, while that in the bread containing WB the SFA remained near the control. On the contrary, the addition of WB increased oleic acid content. With respect to PUFA, the content of linolenic fatty acid was increased when FF or SF was added at higher proportions in breads, being the substitution of 150 g kg^−1^ FF the sample that presented the highest linolenic acid content. The content of the linoleic fatty acid increased to raise the percentage of replacement with SF and decreased in replacement with FF. These results are partially according to Lipilina and Ganji [35], who reported that the incorporation of 150 g kg^−1^ FF produced an increase of 8-fold in linolenic acid (14 g) and a 42.85 % increase in linoleic acid (3 g). The content of CLA (conjugated linoleic acid) declined to values close to zero in most of the samples added with FF. The presence of these fatty acids could be attributed to bovine fat used for making bread. In recent years, these compounds have attracted attention because of their beneficial biological effects, including protection functions against several types of cancer, atherosclerosis, and obesity [36].

Due to the fact that currently, in western diets, there has been an enormous increase in the consumption of n6 fatty acids because of the recommendation to replace saturated fats with n6 fatty acids to reduce concentrations of serum cholesterol [37] the proportion of n6 and n3 of the traditional 1-2 : 1 to 10–20 : 1 [38] has changed. Thus, a diet rich in omega-6 fatty acids shifts the physiological state to one that is prothrombotic and proaggregatory, with increases in blood viscosity and vasoconstriction, and decreases the bleeding time [38]. According to nutritionists, a well-balanced diet should maintain the proportion of fatty acids n6/n3 in a 5 to 10 range or a lower relation [34]. The ratio n6/n3 of different formulations was in accord with the WHO recommendations [34], except loaves with FF which decreased these values to 1.1 and 0.5 (150 g kg^−1^ FF).

The PUFA/SFA relationship of loaves replaced with SF or FF was consistent with the standards set by the Department of Health [34] and showed a ratio greater than 0.45. The loaves with 150 g kg^−1^ FF showed the largest PUFA/SFA ratio. Furthermore, the breads with WB presented the lowest PUFA/SFA relation.

### 3.2. Technological Parameters

Technological characteristics results of breads replaced with different level substitution for FF, SF, and WB were tabulated in Table 4. Loaf volume is considered as one of the most important criteria to evaluate bread quality since it provides a quantitative measurement of baking performance [39]. The replacement of wheat flour by SF, FF, or WB affected the technological quality of bread because the smallest amount of gluten in the samples had repercussions on the specific volume of bread. It can be seen that increasing the substitution significantly reduced the specific volume (*P* < 0.05). No significant differences were observed breads between replaced with 50 g kg^−1^ SF and control. Bread prepared with 150 g kg^−1^ WB had the largest decrease in the specific volume. Gómez et al. [20] found that bran also reduced bread volume. This reduction was observed even with the lowest bran percentage (50 g kg^−1^), whereas, in pan bread, this effect was only observed after the addition of 100 g kg^−1^ bran or more. These differences may be because the pan prevents dough extension (flatting) when dough has low strength. When whole grains begin to replace bread flour, gluten is affected. Gluten is developed from two proteins in flour, gliadin and glutenin, and forms the framework and structure of bread. When hydrated gliadin and glutenin during kneading, these two proteins line up and form a network that traps the CO_2_ produced by the yeast [18]. This might be compensated with the addition of an enhancer or vital wheat gluten. Our results were consistent with studios of Conforti and Davis [18], who found that flax and soya flour caused a decrease in loaf volume. The use of whole wheat flour and ground flaxseed resulted in a larger fiber content, which interfered negatively with the technological quality of bread, especially the reduction of the specific volume in relation to bread prepared with 100% white wheat flour [23].

The moisture content of foods is usually used as an indicator of food quality. It is important to measure the moisture content in bread because of its potential impact on the sensory, physical, and microbial properties of the bread. For example, higher, but ideal moisture, content has been reported to positively increase bread loaf volume [40]. It was observed that samples replaced with WB (50, 100, and 150 g kg^−1^) and 150 g kg^−1^ FF showed significant differences in the values of moisture compared to the control (*P* < 0.05). Breads of WB had the highest moisture level; while bread prepared with 150 g kg^−1^ FF exhibited the lowest moisture level. Moisture content of bread crumb is determined by factors such as flour type, ingredient used, and processing variables [41]. The Argentine Food Code [1] sets a maximum of 310 g kg^−1^ moisture for bread weighing between 100 and 250 g, while international regulations set a 407 g kg^−1^ for traditional bread. Therefore, all samples including control give a moisture level higher than the Argentine regulations but fall within the limits of international regulations.

### 3.3. Crust and Crumb Color

Crust and crumb *L* (light/dark), *a* (red/green) and *b* (yellow/blue) values of breads supplemented with FF, SF, and WB are presented in Table 4. The addition of other flours to bread formulation produced changes in the crumb and crust colour. Therefore, the parameters of crumb and crust color presented significant differences with control. The values *L* ^*^ and *b* ^*^ of crust in breads of FF and WB tended to decrease with the increase of replacement of flours in the formulation. In contrast, the parameter *a* ^*^ increased with the augment of substitution of flours in all breads formulated. Flax breads had darker crust color (lower *L* values). Our results were consistent with Koca and Anil [42], who found that crust colour was darkened with increasing substitution level of flaxseed flour. With increasing FF and WB level, crumb *L* and *b* values decreased but crumb *a* value increased for all formulations. Breads containing flaxseed flour showed a darker and reddish crumb compared with the other samples. Breads with soy presented a more yellow crumb (higher *b* values). This was due to the influence of the original colour of different flours added. Similar results were obtained by Koca and Anil [42] for breads with FF and Alpaslan and Hayta [43] in breads with soy, flaxseed, and corn.

### 3.4. Breadcrumb Texture Profile Analysis

Table 5 shows that ground WB, FF, and SF had a significant (*P* < 0.05) effect on the textural parameters of the samples. The parameters hardness, gumminess, and chewiness increased to raising replacement levels of different flours added. The sample with 150 g kg^−1^ FF produced harder crumb, less cohesiveness, and most gumminess and chewiness. In studies conducted by Conforti and Davis [18], the texture of the control bread was significantly softer than breads containing FF and/or SF. The increase in crumb firmness could also be directly related to an increase in loaf density caused by the low volume in the grain breads [44].

### 3.5. Stress Relaxation of Crumb Bread

Relaxation curves were expressed in dimensionless form using the ratio *F* ^*^ = *F*(*t*)/*F*_0_ where *F*(*t*) is the force at time *t* and *F*_0_ is the initial force before relaxation at 40% compression. Nonlinear regression analysis was used to fit Maxwell's generalized model, which has frequently been used to represent the relaxation phenomenon in several viscoelastic materials, such as gels, fruits, and vegetables [36, 37]. In this case, the model was constituted by a pure elastic element and two Maxwellian elements in parallel and was expressed by the following equation:

(1) $$\begin{array}{c} F^{*}=\frac{F\left(t\right)}{F_{0}}=A_{\infty }+A_{1}\exp\left(\frac{-t}{\lambda_{1}}\right)+A_{2}\exp\left(\frac{-t}{\lambda_{2}}\right), \end{array}$$

where *A*_*∞*_, *A*_1_, and *A*_2_ are constants which depend on the viscoelastic properties of the material and *λ*_1_ and *λ*_2_ are the relaxation times. When (1) is expressed in terms of elastic modulus (*E*) it becomes

(2) $$\begin{array}{c} E\left(t\right)=E_{\infty }+E_{1}\exp\left(\frac{-t}{\lambda_{1}}\right)+E_{2}\exp\left(\frac{-t}{\lambda_{2}}\right). \end{array}$$

*E*_*∞*_ is the equilibrium stress (*σ*_*e*_). The elastic modulus of each element was calculated as follows:

(3) $$\begin{array}{c} E_{i}=\frac{A_{i}\times F_{0}}{\left(\varepsilon_{t}\times a_{0}\right)}, \end{array}$$

where *A*_*i*_ are the coefficients in (2) and *ε*_*t*_ is the true deformation at the beginning of relaxation. Relaxation times were related to the viscosity coefficient (Pascals × second) in Maxwell's model according to Kfoury et al. [45]:

(4) $$\begin{array}{c} \eta_{i}=E_{i}\times \lambda_{i}. \end{array}$$

The parameter values (*E*_*i*_ and *η*_*i*_) are shown in Table 6. The elastic modulus and viscosity coefficient are related with hardness and/or springiness. When the elastic modulus increases, the crumb becomes harder. While the viscous modulus increases, the crumb is less elastic. Both parameters were increased to raising replacement levels of different flours added. Therefore, crumb breads with 150 g kg^−1^ were harder and less springiness, which correlates with the results of TPA.

Correlation analysis showed that crumb hardness, chewiness, and gumminess were most strongly positive correlated with the relaxation parameters of breads (Table 7). Therefore, crumb springiness and cohesiveness presented negative correlation with the relaxation parameters of breads. With an increase of replacement levels of different flours added, there was increase in the hardness and a decrease of springiness of bread crumb. This demonstrates that empirical textural characteristics (hardness and springiness) were consistent with fundamental mechanical properties (*E*_*i*_ and *η*_*i*_) of replaced bread.

### 3.6. Sensorial Analysis

**Figure 1:**
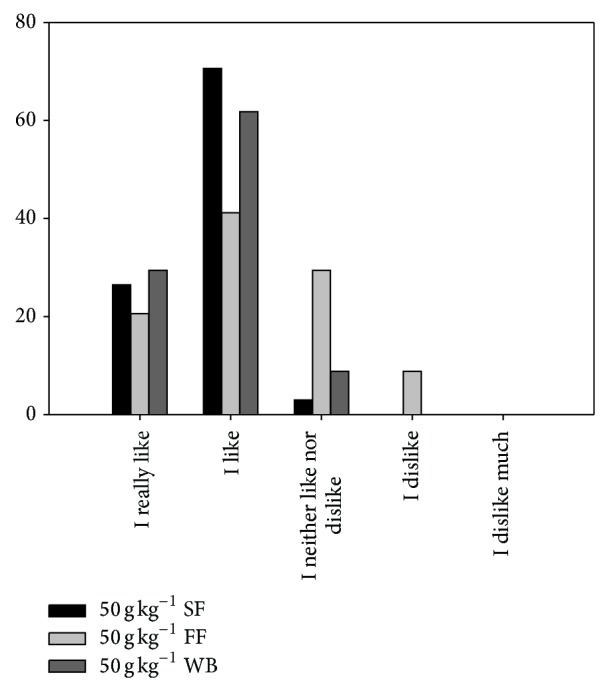
Results of sensorial analysis of selected bread. SF: soybean flour, FF: flaxseed flour, and WB: wheat bran.

The formulations with 50 g kg^−1^ of SF, FF, or WB were selected for good specific volume (similar control) and less hard and more springiness crumb.  Figure 1 shows the results of sensorial analysis of selected bread.

The selected breads with SF and WB were well accepted with an overall linking score of 4.24 and 4.21, respectively (in a scale of 5). More than 90% of the evaluators reported the total acceptability of the bread with SF and WB, stating that they liked for the taste and texture. However the rest of judges expressed that they neither like nor dislike the product, although they also would consume. On the other hand, the bread with 50 g kg^−1^ FF showed moderated acceptance with an overall linking score of 3.74. Also, the 41.18% of panelist opined that they liked the bread with FF for its good texture but possessed a bitter residual taste, whereas the 20.9% of the evaluators indicated that they like much this bread for its crunchy crust and fluffy crumb. Nevertheless, the 29.41% of judges expressed that they neither like nor dislike the product due intense bitter taste. This is according to Menteş et al. [27] who informed that all flaxseed supplementation breads were found acceptable.

## 4. Conclusions

The addition of these functional flours produced an improvement of fatty acids composition. As a result, FF or SF produced a decrease in SFA and MU content and an increase of PUFA in these breads. Thus, linoleic fatty acid was increased to intensify the level of SF and decreased with replacement of FF. Furthermore, breads replaced with FF presented considerable increase in the content of n3 fatty acids and consequently, the proportion of n6/n3 was reduced considerably, while the addition of WB did not decrease the SFA content but raised oleic fatty acid content. The substitution percentage increase of FF, SF, or WB to formulation produced changes in the colour, rheological, textural, and technological characteristics of breads, giving darker crumb and crust, less springriness, and harder crumb and produced a decrease of specific volume breads.

The breads with 50 g kg^−1^ soy flour were those with better technological and rheological features, very good sensorial acceptance, and improved nutritional profile of fatty acids. The results showed the possibility of development of one nutritionally safe product with sensory acceptance. The study of bread with soy, as well as the association with other flour as flaxseed in the same product, could enable the development of a range of functional products.

## Figures and Tables

**Table 1:** Composition of fatty acids (g kg^−1^ of total fatty acids) of different flours (WF, SF, FF, and WB)^a^ and BF.

Fatty acids	WF	SF	FF	WB	BF
(14:0)	nd	nd	nd	nd	25.9
(15:0)	nd	nd	nd	nd	6.33
(16:0)	165.3^d^	99.4^b^	60.7^a^	151.1^c^	246.92
(16:1)	nd	nd	nd	nd	28.11
(17:0)	nd	nd	nd	nd	13.68
(18:0)	15.9^b^	46^c^	43.3^c^	12.8^a^	254.39
(18:1)c n9	145.2^a^	197.9^c^	160.8^b^	167.9^b^	323.5
(18:1)t vaccenico	nd	nd	nd	nd	43.05
(18:2)c n6	628.8^c^	576.1^b^	145.7^a^	615.5^c^	32.32
(20:0)	nd	nd	nd	nd	4.92
(20:1) + (18:3) n3	43.5^a^	79^c^	580.9^d^	51.3^b^	9.39
CLA (18:2) 9c, 11t	nd	nd	nd	nd	11.3

SFA	181.9^d^	147.1^b^	104^a^	163.9^c^	552.14
MU	145.2^a^	197.9^c^	160.8^ab^	167.9^b^	394.66
PUFA	674.8^a^	654^a^	725^b^	665^a^	41.71
n3	43.5^a^	79.9^c^	580.9^d^	51.3^b^	9.39
n6	628.8^c^	576.1^b^	145.7^a^	615.5^c^	32.32
n9	145.2^a^	197.9^c^	160.8^ab^	167.9^b^	323.5
n6/n3	14.46^d^	7.22^b^	0.25^a^	12.01^c^	3.4
PUFA/SFA	0.371^a^	0.446^c^	0.69^d^	0.407^b^	0.075

^a^Mean values with different letters in a column are significantly different (*P* < 0.05). nd: not detected, SFA: summation of saturated fatty acids, MU: summation of monounsaturated fatty acids, PUFA: summation of polyunsaturated fatty acid, PUFA/SFA: relationship of polyunsaturated fatty acid and saturated fatty acids, n3: fatty acids of the n3 family, n6: fatty acids of the n6 family, n9: fatty acids of the n9 family, n6/n3: relationship between fatty acids of series n6 and n3, WF: wheat flour, SF: soybean flour, FF: flaxseed flour, WB: wheat bran, and BF: bovine fat.

**Table 2:** Fatty acid composition (g kg^−1^ of total fatty acids) of loaves replaced with SF, FF, and WB (50, 100, and 150 g kg^−1^) and control^a^.

Replaced breads	(14:0)	(15:0)	(16:0)	(16:1)	(17:0)	(18:0)	(18:1)t	(18:1)c	(18:2)c	(20:0)	*α*(18:3) n3	CLA
Control	24.2^gh^	5.7^i^	237.1^h^	18.9^e^	11.5^ef^	214.1^g^	22.7^i^	264.1^g^	173.1^g^	nd	18.8^c^	11.2^cd^
50 g kg^ −1^ SF	21.2^f^	4.7^e^	211.1^f^	19.8^f^	11.1^e^	186.1^f^	18.6^f^	252.1^f^	232.1^h^	2.8^ab^	27.7^d^	9.8^b^
100 g kg^ −1^ SF	17.5^d^	4^d^	197.1^e^	16.5^d^	9.6^d^	167.1^d^	15.9^d^	242.1^e^	284.1^i^	2.7^ab^	33.8^e^	8.8^a^
150 g kg^ −1^ SF	16.3^c^	3.9^c^	189.1^c^	15.6^c^	8.2^b^	159.1^c^	14.3^c^	239.1^c^	303.1^j^	2.8^ab^	38.2^f^	8.7^a^
50 g kg^ −1^ FF	19.2^e^	4.9^f^	194.1^d^	15.2^c^	8.8^c^	173.1^e^	17.2^e^	240.1^d^	170.1^f^	nd	158.1^g^	nd
100 g kg^ −1^ FF	14.9^b^	3.5^b^	166.1^b^	14^b^	7.9^b^	149.1^b^	13.2^b^	227.1^b^	154.1^d^	2.3^a^	249.1^h^	nd
150 g kg^ −1^ FF	13.7^a^	3^a^	154.1^a^	11.2^a^	6.5^a^	138.1^a^	12.1^a^	220.1^a^	146.1^c^	nd	296.1^i^	nd
50 g kg^ −1^ WB	24.5^h^	5.7^i^	240.1^i^	24.9^h^	14.2^h^	240.1^j^	24.9^j^	267.1^h^	133.1^a^	3^b^	17.3^ab^	10.9^c^
100 g kg^ −1^ WB	23.9^g^	5.6^h^	233.1^g^	22^g^	12.8^g^	225.1^i^	20.6^g^	268.1^i^	159.1^e^	2.7^ab^	17.1^a^	11.2^cd^
150 g kg^ −1^ WB	24.6^h^	5.2^g^	237.1^h^	19.2^e^	11.7^f^	217.1^h^	21.7^h^	274.1^j^	142.1^b^	2.8^ab^	17.7^b^	11.3^d^

^a^Mean values with different letters in a column are significantly different (*P* < 0.05). nd: not detected, WF: wheat flour, SF: soybean flour, FF: flaxseed flour, and WB: wheat bran.

**Table 3:** Total contents of SFA, MU, PUFA, n3, n6, n9 (g kg^−1^ of total fatty acids) and relationship of PUFA/SFA and n6/n3 of loaves replaced with SF, FF, and WB (50, 100, and 150 g kg^−1^) and control^a^.

Replaced breads	SFA	MU	PUFA	n3	n6	n9	n6/n3	PUFA/SFA
Control	492.1^g^	283.1^g^	192.1^d^	18.8^c^	173.1^g^	264.1^g^	9.2^f^	0.39^d^
50 g kg^ −1^ SF	437.1^f^	275.1^f^	260.1^e^	27.7^d^	232.1^h^	252.1^f^	8.38^e^	0.6^e^
100 g kg^ −1^ SF	397.1^d^	261.1^e^	318.1^f^	33.8^e^	284.1^i^	242.1^e^	8.41^e^	0.8^f^
150 g kg^ −1^ SF	380.1^c^	256.1^d^	341.1^h^	38.2^f^	303.1^j^	239.1^c^	7.93^d^	0.9^h^
50 g kg^ −1^ FF	400.1^e^	255.1^c^	328.1^g^	158.1^g^	170.1^f^	240.1^d^	1.08^b^	0.82^g^
100 g kg^ −1^ FF	343.1^b^	241.1^b^	403.1^i^	249.1^h^	154.1^d^	227.1^b^	0.62^a^	1.17^i^
150 g kg^ −1^ FF	315.1^a^	231.1^a^	442.1^j^	296.1^i^	146.1^c^	220.1^a^	0.49^a^	1.4^j^
50 g kg^ −1^ WB	529.1^j^	295.1^h^	161.1^b^	17.3^ab^	159.1^e^	267.1^h^	7.69^c^	0.3^a^
100 g kg^ −1^ WB	506.1^i^	298.1^i^	176.1^c^	17.1^a^	133.1^a^	268.1^i^	9.3^f^	0.36^c^
150 g kg^ −1^ WB	495.1^h^	301.1^j^	159.1^a^	17.7^b^	142.1^b^	274.1^j^	8.03^d^	0.31^b^

^a^Mean values with different letters in a column are significantly different (*P* < 0.05). SFA: summation of saturated fatty acids, MU: summation of monounsaturated fatty acids, PUFA: summation of polyunsaturated fatty acid, PUFA/SFA: relationship of polyunsaturated fatty acid and saturated fatty acids, n3: fatty acids of the n3 family, n6: fatty acids of the n6 family, n9: fatty acids of the n9 family, n6/n3: relationship between fatty acids of series n6 and n3, WF: wheat flour, SF: soybean flour, FF: flaxseed flour, and WB: wheat bran.

**Table 4:** Technological and color parameters of breads replaced with 50, 100, and 150 g kg^−1^ of functional flours^a^.

Replaced breads	Technological parameters		Crust color			Crumb color		
	Specific volume (cm^3^ g^−1^)	Moisture (g kg^−1^)	*L* ^*^	*a* ^*^	*b* ^*^	*L* ^*^	*a* ^*^	*b* ^*^
Control	4.85^g^	335.6^b^	54.67^abc^	15.33^c^	21.2^bc^	68.27^e^	2.7^a^	10.73^ab^
50 g kg^−1^ SF	4.63^fg^	336.4^b^	54.63^abc^	9.1^a^	16.43^a^	67.07^de^	4.3^b^	13.63^c^
100 g kg^−1^ SF	4.58^f^	329.9^ab^	56.37^bc^	11.77^ab^	16.57^a^	68.03^e^	4.9^bcd^	16.07^d^
150 g kg^−1^ SF	3.38^ab^	336.9^b^	58.27^cd^	13.53^bc^	18.03^ab^	69.9^e^	4.47^bc^	16.83^d^
50 g kg^−1^ FF	3.9^e^	334^b^	61.03^de^	8.63^a^	18.17^ab^	62.27^bcd^	4.1^b^	10.9^a^
100 g kg^−1^ FF	3.8^de^	318.5^a^	53.37^ab^	9.07^a^	15.43^a^	58.8^ab^	5.23^cd^	10.6^a^
150 g kg^−1^ FF	3.56^bc^	334.5^b^	51.13^a^	9.4^a^	14.67^a^	56.73^a^	5.6^d^	10.43^a^
50 g kg^−1^ WB	3.65^cd^	353^c^	64.03^e^	11.43^ab^	23.43^c^	65.1^cde^	4.47^bc^	12^b^
100 g kg^−1^ WB	3.28^a^	355.5^cd^	60.43^de^	12.77^bc^	23.13^c^	62.37^bcd^	4.9^bcd^	12.57^bc^
150 g kg^−1^ WB	3.57^bcd^	365^d^	57.3^cd^	13.4^bc^	21.53^bc^	61.6^abc^	5.59^d^	13.47^c^

^a^Mean values with different letters in a column are significantly different (*P* < 0.05). WF: wheat flour, SF: soybean flour, FF: flaxseed flour, and WB: wheat bran.

**Table 5:** Texture parameters of loaves replaced with FF, SF, and WB (50, 100, and 150 g kg^−1^) and control^a^.

Replaced breads	Hardness (N)	Adhesiveness (J)	Cohesiveness	Springiness (mm),	Chewiness (mJ)	Gumminess (N)
Control	1.08^a^	0.07^a^	0.54^d^	9.92^ab^	5.7^a^	0.58^a^
50 g kg^−1^ SF	3.45^c^	0.07^a^	0.5^cd^	8.49^a^	14.67^bcd^	1.74^bc^
100 g kg^−1^ SF	4.67^d^	0.1^a^	0.44^abc^	8.61^ab^	17.75^d^	2.06^cd^
150 g kg^−1^ SF	5.73^e^	0.09^a^	0.5^bcd^	8.82^ab^	25.16^e^	2.85^e^
50 g kg^−1^ FF	3.16^c^	0.08^a^	0.45^abc^	7.94^a^	11.19^abc^	1.41^b^
100 g kg^−1^ FF	5.59^e^	0.07^a^	0.42^ab^	8.13^a^	19.13^d^	2.35^d^
150 g kg^−1^ FF	6.04^e^	0.11^a^	0.4^a^	7.04^a^	16.92^d^	2.4^d^
50 g kg^−1^ WB	1.41^ab^	0.06^a^	0.54^d^	12.52^b^	9.88^ab^	0.76^a^
100 g kg^−1^ WB	1.95^b^	0.08^a^	0.47^abcd^	9.59^ab^	8.74^a^	0.91^a^
150 g kg^−1^ WB	3.46^c^	0.06^a^	0.47^abcd^	10.27^ab^	15.93^cd^	1.61^b^

^a^Mean values with different letters in a column are significantly different (*P* < 0.05). WF: wheat flour, SF: soybean flour, FF: flaxseed flour, and WB: wheat bran.

**Table 6:** Relaxation parameters of loaves replaced with FF, SF, and WB (50, 100, and 150 g kg^−1^) and control^a^.

Replaced breads	E_∞_ (kPa)	E_1_ (kPa)	E_2_ (kPa)	η_1_ (Pa*·*s)	η_2_ (Pa*·*s)	R^2^
Control	3.42^a^	1.52^a^	64.64^abc^	141.43^a^	42.42^b^	0.9993
50 g kg^−1^ SF	5.75^bc^	2.70^b^	61.40^abc^	234.24^bc^	27.04^a^	0.9998
100 g kg^−1^ SF	9.64^d^	4.71^d^	152.60^f^	399.22^d^	35.14^ab^	0.9998
150 g kg^−1^ SF	12.27^e^	5.51^e^	111.11^de^	513.65^e^	24.77^a^	0.9997
50 g kg^−1^ FF	6.94^c^	3.48^c^	88.93^cd^	286.81^c^	30.12^a^	0.9998
100 g kg^−1^ FF	8.87^d^	4.73^d^	140.66^ef^	397.55^d^	34.12^ab^	0.9998
150 g kg^−1^ FF	9.33^d^	4.99^de^	124.45^ef^	412.43^d^	31.94^ab^	0.9998
50 g kg^−1^ WB	3.49^a^	1.67^a^	38.73^a^	133.57^a^	29.31^a^	0.9998
100 g kg^−1^ WB	4.42^ab^	2.04^a^	45.88^ab^	167.23^ab^	27.29^a^	0.9992
150 g kg^−1^ WB	6.20^c^	2.96^bc^	78.21^bcd^	238.85^bc^	30.13^a^	0.9999

^a^Mean values with different letters in a column are significantly different (*P* < 0.05). WF: wheat flour, SF: soybean flour, FF: flaxseed flour, and WB: wheat bran.

**Table 7:** Pearson's correlation coefficients between relaxation and texture parameters of loaves replaced with FF, SF, and WB (50, 100, and 150 g kg^−1^) and control.

	*σ*_*e*_	*E*_1_ (kPa)	*E*_2_ (kPa)	*n*_1_	*n*_2_	Hardness (N)	Adhesiveness (J)	Cohesiveness	Springiness (mm)	Chewiness (mJ)	Gumminess (N)
*σ*_*e*_	1										
*E*_1_ (kPa)	0.98^*^	1									
*E*_2_ (kPa)	0.79^*^	0.85^*^	1								
*n*_1_	0.99^*^	0.99^*^	0.81^*^	1							
*n*_2_	—	—	—	—	1						
Hardness (N)	0.91^*^	0.95^*^	0.82^*^	0.92^*^	—	1					
Adhesiveness (J)	—	—	—	—	—	—	1				
Cohesiveness	—	−0.6^*^	−0.6^*^	−0.52^*^	—	−0.66^*^	—	1			
Springiness (mm)	−0.5^*^	−0.54^*^	−0.55^*^	−0.51^*^	—	−0.54^*^	—	0.48^*^	1		
Chewiness (mJ)	0.87^*^	0.85^*^	0.65^*^	0.86^*^	—	0.88^*^	—	−0.37^*^	—	1	
Gumminess (N)	0.93^*^	0.94^*^	0.77^*^	0.94^*^	—	0.98^*^	—	−0.51^*^	−0.5^*^	0.94^*^	1

^*^Significant at *P* < 0.05.
